# Computer-aided endoscopic sinus surgery: a retrospective comparative study

**DOI:** 10.4103/0256-4947.60522

**Published:** 2010

**Authors:** Jamil N. Al-Swiahb, Surayie H. Al Dousary

**Affiliations:** From the Department of Otorhinolaryngology, Head and Neck Surgery, King Abdulaziz University Hospital, Riyadh, Saudi Arabia

## Abstract

**BACKGROUND AND OBJECTIVES:**

Endoscopic sinus surgery (ESS), markedly improved with the introduction of new preoperative imaging techniques, intraoperative visualization tools, and the use of surgical navigation systems. In this retrospective study we evaluated the usefulness of CT-guided endscopic sinus surgery and studied its advantages over conventional endscopic sinus surgery.

**METHODS:**

We retrospectively reviewed the records of 60 randomly chosen patients with chronic rhinosinusitis (CRS) and moderate-to-severe sinonasal polyposis, undergoing endoscopic sinus surgery with surgical navigation (n=30) and without navigation (n=30). Data on the operative note, time of surgery, complications, and recurrence rate were collected and analyzed.

**RESULTS:**

Of the 60 patients, 40 (66.7%) were diagnosed with CRS and 20 (33.3%) had allergic fungal sinusitis. Primary surgery was performed in 37 (61.7%) and revision surgery was performed in 23 (38.3%) cases. The computer-aided surgery (CAS) group included 28 (93.3%) patients with extensive disease and 12 (40%) with bone erosions, with intraorbital or extradural extension, while the non-CAS group included 24 (80%) patients with extensive disease and seven (23.3%) with bone erosions, with intraorbital or extradural extension. The average operative time was approximately 13 minutes greater in the navigation group, with significant improvement in the recurrence rate (n=11, 36.7% in the non-CAS group; n=5, 16.7% in the CAS group), and intraoperative complications were fewer in the CAS group (two exposures of orbital fat in the non-CAS group; no complications in the CAS group).

**CONCLUSION:**

Computer navigational systems appear to serve as a valuable adjunct in preoperative planning and safe intraoperative dissection.

Endoscopic sinus surgery (ESS) is a treatment of choice for chronic rhinosinusitis (CRS), with an expanding role in the management of other sinus, orbit, and skull base diseases. Despite the availability of excellent nasal telescopes and high-resolution preoperative computed tomography (CT), major complications such as blindness, central nervous system (CNS) trauma, and even death still occur, because the ESS may be compromised by anatomic complexity or intraoperative bleeding.^1^ Of late, computer-aided surgery (CAS) technology has permitted a direct comparison of the intraoperative anatomy with preoperative imaging information.^2^ After a registration and calibration process, the surgeon may point to a specific structure with the CAS instrument and then view the location of the instrument tip on the CT image.^3^ The use of CAS systems may allow for more precise dissections and greater rates of sinus patency outcomes and fewer complications.^2^–^4^ We describe our experience with CAS as applied to intraoperative guidance and compare it with surgery without imaging guidance, in terms of safety of surgery, duration, complications, and outcome.

## METHODS

We retrospectively studied 60 patients with CRS who underwent endoscopic sinus surgery between January 1, 2000 and 30 December 2006, including 30 patients who had conventional endoscopic sinus surgery and 30 patients who had endoscopic sinus surgery with use of the navigational system. The LandmarX system (Medtronic-Xomed) was used for the image-guided cases. The presence of extensive disease (stage III-IV nasal polyposis) was based on the Lund-Mackay endscopic grade system (polyps were scored as grade I when restricted to the middle meatus, grade II when they reached beyond the middle turbinate, grade III when they reached the inferior turbinate, and grade IV when filled the nasal cavity). Moreover, the total score was more than 12 in the Lund-Mackay CT scan classification system of CRS (The Lund-Mackay CT scan grading system relied on a score of 0-2, depending on the absence, partial or complete opacification of each sinus system, and of the ostiomeatal complex). Patients who had external sinus surgery or who were not diagnosed as having chronic rhinosinusitis were excluded. Before surgery, a standardized CAS sinus CT scan (1 mm axial image) was obtained and transferred to the CAS workstation, where coronal and sagittal images, in addition to 3D-model reconstructions and a software-based CT review was completed.

After induction of general anesthesia, a headset that permitted attachment of a reference frame, with light-emitting diodes for optical tracking, was placed securely on the patient's head and registration and calibration was performed. All patients underwent endoscopic ethmoidectomy, endoscopic maxillary antrostomy, endoscopic sphenoidectomy, and endoscopic frontal recess exposure with frontal sinus suction clearance. The CAS probes were used frequently for confirmation of the surgical tool position. Usually we kept most of the patients in the hospital for one or two days, with discharge, and followed up after one week, when endoscopic debridement was performed. Patients were then followed up every two weeks until the healing process was complete, and then every two months with local treatment. The various parameters compared descriptively between the two groups of patients included, patient demographics, surgical details, operative times, the incidence of complications, and the outcome.

## RESULTS

The study population included 38 males (63.3%) and 22 females (36.7%), with a mean age of 28.2 years (range 11-63 years). All the patients underwent ESS, for a diagnosis of CRS in 40 patients (66.7%) and allergic fungal sinusitis (AFS) in 20 patients (33.3%). Surgery was primary in 37 patients (61.7%), and revision in 23 patients (38.3%). The clinical characteristics of patients in both groups were almost similar, with most of the patients presenting with nasal obstruction and nasal discharge hyposmia. Six (20%) in the CAS group had proptosis and one had diplopia; in the non-CAS group, seven (23.3%) had proptosis. Additional comorbidities included allergic rhinitis in 54 patients (90%), nasoseptal deviation in 38 (63.3%), and bronchial asthma in 20 (33.3%). Nasal polyps were graded III in 12 (40%) and IV in 24 (80%) patients in the non-CAS group, while in the CAS group, polyps were graded III in 12 (40%) and IV in 28 (93.3%) patients. Most patients had similar surgeries and pre- and postoperative management. Comparison of CT scan finding was performed (Table 1). The mean time of surgery was 3 hours and 26 minutes in the CAS group and 3 hours and 13 minutes in the non-CAS group. A higher recurrence rate was seen in the non-CAS group (11 patients, 36.7%) compared to the CAS group (five patients, 16.7%) in the postoperative period ranging from one to two years. Five patients (16.7%) from the non-CAS group needed revision surgery compared to two patients (6.8%) from the CAS group. Intraoperative complications did not occur in any of the patients in the CAS group and exposure of orbital fat occured in two patients in the non-CAS group. The incidence of postoperative complications was similar for both the groups. Two patients in each group had secondary bleeding; one was treated conservatively and other had to be controlled under general anesthesia. Two patients developed adhesions that were released under local anesthesia. Three patients from the CAS group reported persistent headache in the first postoperative week, which resolved completely with analgesics.

**Table 1:** CT scan findings in both groups.

	Non-CAS surgery (%)	CAS surgery (%)
Maxillary opacity	30 (100)	30 (100)
Ethmoid sinuses opacity	30 (100)	30 (100)
Frontal sinus opacity	16 (53.3)	26 (86.7)
Sphenoid sinuses opacity	19 (63.3)	29 (96.7)
Expansion effect	12 (40)	14 (46.2)
Bony wall erosion	7 (23.3)	12 (40)
Intraorbital extension	4 (13.3)	11 (36.7)
Extradural extension	3 (10)	11 (36.7)

CAS: Computer-aided surgery

## DISCUSSION

Otorhinolaryngologic surgery in the region near the skull base must be safe and thorough. Most complications occur if the surgeon is not aware of the exact position of his instrument. On account of that, interest in the use of image guidance systems in otolaryngology increased with the development of ESS.^3^,^4^ At present, electromagnetic and optical systems are the most widely used because of their acceptable accuracy to within 2 mm or less, the freedom of head movement, and the ability to track a variety of instruments.^1^,^4^–^7^ In our department, we use the LandmarX system (Medtronic-Xomed) and we are the first university in Saudi Arabia using this technology. The indications for use of the navigation system are usually extensive disease or previous surgery with poor anatomic landmarks.^8^ The navigation system is used to reduce complication rates, completely remove the disease, and hasten the recovery after surgery.

**Figure 1:**
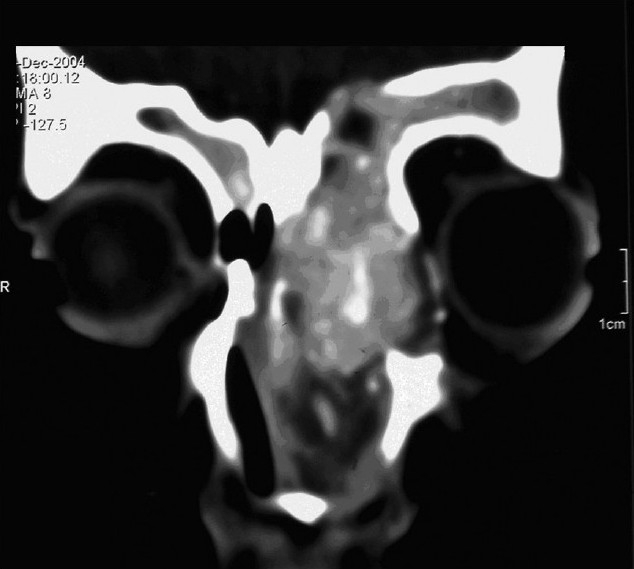
Fifteen years with allergic fungal sinusitis with intraorbital and extradural extension.

**Figure 2:**
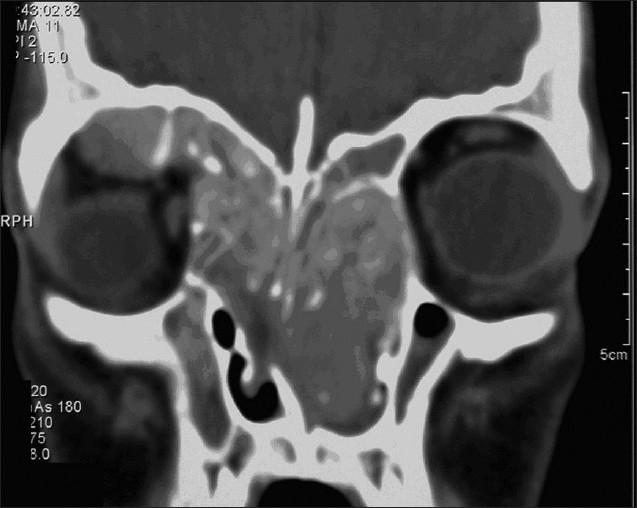
Sixteen years with supraorbital and intraorbital extension

CAS is most helpful in specific anatomic areas, especially in the frontal sinus,^8^ the sphenoid and the sphenoethmoid regions, the residual ethmoid partition and disease, in skull base identification, and in orbital dehiscence or orbital surgery for optic nerve or orbital decompression.^2^,^3^,^9^ We have many difficult cases that cannot be done safely without this technology (for example, in those who have had previous surgery with distorted anatomy or those with bone erosions, and intraorbital or extradural extension (Figures 1 and 2).

Hepworth and colleagues performed surveys in the US and found that the most commonly acceptable indications for CAS were revision surgery, in 84%, followed by frontal and sphenoid surgery; and they agreed that image-guided surgery should become the standard of care for functional endoscopic sinus surgery.^3^,^4^,^8^ Olson and Citardi found that the most common diagnoses that indicated the use of CAS were chronic frontal sinusitis (92%), previous surgery (81%), sinonasal polyposis (48%), AFS (3%), chronic granulomatous fungal sinusitis (2%), cystic fibrosis (2%), and previous maxillofacial trauma (2%).^2^ In our group, we found that chronic rhinosinusitis with sinonasal polyposis was the most common diagnosis (67%) and approximately 46% had frontal sinusitis in the CAS group, and 33.3% with AFS, because this sinonasal problem was common in our area. Revision surgery was required in 38.3%.

Almost all of our patients underwent endoscopic ethmoidectomy, endoscopic maxillary antrostomy, endoscopic sphenoidectomy, and endoscopic frontal recess surgery. We found that CAS was valuable in confirming the surgeon's understanding of the paranasal sinus anatomy, and helpful in performing a more complete anatomic dissection in patients with extensive disease or heavy intraoperative bleeding, which helped to reduce intraoperative complications and provide better outcomes.^4^,^7^ Also, we found it a valuable teaching tool in the residency training program in our institute.^3^,^7^,^10^ Intraoperative CAS registration accuracy was estimated to be 2 mm or better.^1^,^6^ To determine system accuracy, we compared the CAS instrument tip position indicated by the CAS system with its actual location in the operative field at several areas of the operative volume.^6^ The estimated accuracy represents the surgeon's visual estimate of system accuracy.^7^

As outlined by Roth et al, CAS should provide an accuracy from 2 to 3 mm.^1^,^11^ The system was used only if the perceived accuracy was < 3 mm; if the drift was >3 mm the device was not to be used for localization. The device was verified at intervals ranging from 15 to 45 minutes.^3^,^9^ In our group, we found the device was accurate up to 2 mm, by visually estimating the device probe to well-known visible anatomical landmarks. If it were not to this level, we reverified the device, to improve the accuracy. The duration of registration was approximately 15-30 minutes.

Initial use of the CAS has been found to increase the operative time by 15 to 30 minutes. Once a surgeon becomes familiar with the equipment, this time is typically reduced, from 5 to 15 minutes.^12^ We have found that the operative time increases with CAS to approximately 10 to 20 minutes, with an average of 13 minutes, because of equipment manipulation and registration, but real time in surgery is reduced compared to non-CAS, because of improvement in the surgeon's confidence and knowledge of patient anatomy, which allows the surgeon to be more efficient while removing the pathology.^4^,^7^,^13^

Intraoperative image guidance is expected to increase safety and decrease complications during ESS.^2^,^6^ When a surgeon is well versed in the sinus anatomy, in general, and in the individual patient's anatomy in particular, use of this technology to confirm the position within the sinuses, probably enhances the safety.^6^,^13^ Major complications from sinus surgery are relatively infrequent.^1^,^2^,^14^ The impact of image-guided technology on the complication rate has not been evaluated.^12^

In our patients, we found that the recurrence rate and complications were reduced even though the number of patients was not large enough to relatively demonstrate the outcome. In the CAS group, three patients complained of a persistent, moderate-to-severe headache, which was completely resolved with analgesics. The cause of the headache appeared to be due to the pressure caused by a tight-fitting headset. Wang and Maccabees reported six patients with facial neuropathies (both sensory and motor) related to the use of a headset.^15^

The future applications for image-guided surgeries are broad. They include otology, skull-base surgery,^13^ and craniofacial surgery, as well as maxillofacial trauma.^7^ In 1996, Fried et al. described the potential for using an MRI scanner for ESS in a suite that permitted ‘real time’ images and was set-up for operative procedures.^16^ Klapan et al. designed a 3D-computer-assisted functional ESS for presurgical planning, intraoperative guidance, and postoperative analysis of the anatomic regions of the nose and paranasal sinuses.^14^ Still, the technology is a promising avenue of research in the field of image-guidance for otolaryngology.^3^

In conclusion, the use of computer assistance is firmly established as a valuable technology in the management of paranasal sinus disease. It may improve the confidence of the surgeon by confirming the position within the challenging anatomic fields. This serves to increase surgical effectiveness and decrease surgical morbidity. The major drawback of using image-guided systems is the increased operative time, which improves with advancement in technology. The effect of CAS on the clinical outcome is yet to be demonstrated.
